# Cystatin C Confirms the Canagliflozin eGFR Slope Benefit in CANPIONE

**DOI:** 10.1016/j.ekir.2026.106374

**Published:** 2026-02-23

**Authors:** Satoshi Miyamoto, Hiddo J.L. Heerspink, Dick de Zeeuw, Kota Sakamoto, Michihiro Yoshida, Masao Toyoda, Daisuke Suzuki, Takashi Hatanaka, Tohru Nakamura, Shinji Kamei, Satoshi Murao, Kazuyuki Hida, Shinichiro Ando, Hiroaki Akai, Yasushi Takahashi, Munehiro Kitada, Hisashi Sugano, Tomokazu Nunoue, Akihiko Nakamura, Motofumi Sasaki, Tatsuaki Nakatou, Kei Fujimoto, Daiji Kawanami, Takashi Wada, Nobuyuki Miyatake, Hiromi Kuramoto, Kenichi Shikata

**Affiliations:** 1 Center for Innovative Clinical Medicine, Okayama University Hospital, Okayama, Japan; 2 Department of Internal Medicine, Innoshima General Hospital, Onomichi, Japan; 3 Department of Clinical Pharmacy and Pharmacology, University of Groningen, University Medical Center Groningen, Groningen, The Netherlands; 4 Division of Nephrology, Endocrinology and Metabolism, Department of Internal Medicine, Tokai University School of Medicine, Isehara, Japan; 5 Suzuki Diabetes Clinic, Atsugi, Japan; 6 Department of Diabetes and Endocrinology, National Hospital Organization Fukuyama Medical Center, Fukuyama, Japan; 7 Diabetes Internal Medicine, Sumitomo Besshi Hospital, Nihama, Japan; 8 Department of Diabetic Medicine, Kurashiki Central Hospital, Kurashiki, Japan; 9 Department of Diabetes and Endocrinology, Takamatsu Hospital, Takamatsu, Japan; 10 Department of Diabetology, Metabolism and Endocrinology, National Hospital Organization Okayama Medical Center, Okayama, Japan; 11 Department of Internal Medicine Diabetic Center, Okayama City Hospital, Okayama, Japan; 12 Division of Diabetes and Metabolism, Faculty of Medicine, Tohoku Medical and Pharmaceutical University, Sendai, Japan; 13 Department of Diabetes, Ochiai General Hospital, Maniwa, Japan; 14 Department of Diabetology and Endocrinology, Kanazawa Medical University, Uchinada, Ishikawa, Japan; 15 Division of Anticipatory Molecular Food Science and Technology, Medical Research Institute, Kanazawa Medical University, Uchinada, Ishikawa, Japan; 16 Department of Diabetes and Endocrinology, Kochi Health Sciences Center, Kochi, Japan; 17 Nunoue Clinic, Tsuyama, Japan; 18 Internal Medicine, Osafune Clinic, Setouchi, Japan; 19 Department of Diabetes and Endocrinology, Matsue City Hospital, Matsue, Japan; 20 Diabetes Center, Okayama Saiseikai General Hospital, Okayama, Japan; 21 Division of Diabetes, Metabolism and Endocrinology, Department of Internal Medicine, The Jikei University Kashiwa Hospital, Kashiwa, Japan; 22 Department of Endocrinology and Diabetes, Fukuoka University School of Medicine, Fukuoka, Japan; 23 Department of Nephrology and Rheumatology, Graduate School of Medical Sciences, Kanazawa University, Kanazawa, Japan; 24 Department of Hygiene, Faculty of Medicine, Kagawa University, Miki, Kagawa, Japan

**Keywords:** chronic kidney disease, cystatin C, eGFR slope, microalbuminuria, SGLT2 inhibitor, type 2 diabetes

## Abstract

**Introduction:**

In the canagliflozin in type 2 diabetic patients with microalbuminuria in Japanese population (CANPIONE) study with a novel preintervention slope design, canagliflozin attenuated the decline in creatinine-based estimated glomerular filtration rate (eGFR-creatinine) slope in patients with type 2 diabetes mellitus and microalbuminuria. However, because serum creatinine is affected by muscle mass, the reliability of the eGFR-creatinine slope may be limited. Therefore, this prespecified exploratory analysis aimed to evaluate the effect of canagliflozin on the cystatin C-based eGFR (eGFR-cystatin C) slope, which is less affected by muscle mass, to validate the robustness of the previous findings.

**Methods:**

The chronic eGFR-cystatin C and eGFR-creatinine slopes were assessed using a 2-slope linear spline mixed-effects model. Pearson correlation coefficients were used to evaluate associations between changes in eGFR and body composition-related parameters.

**Results:**

eGFR trajectories were directionally consistent across both filtration markers. The chronic eGFR-cystatin C slopes in the canagliflozin and control groups were −0.4 (95% confidence interval [CI], −2.6 to 1.8) and −4.9 (−7.1 to −2.6) ml/min per 1.73 m^2^/year, respectively, corresponding to a between-group difference of 4.4 (1.3–7.6) ml/min per 1.73 m^2^/year. Similar trends were observed in the eGFR-creatinine slopes derived from the same time points, yielding a comparable treatment effect on the chronic slope (between-group difference: 3.5 [0.2–6.8] ml/min per 1.73 m^2^/year). Canagliflozin reduced body weight, body mass index (BMI), and waist circumference, whereas little correlation was observed between these changes and changes in eGFR-cystatin C.

**Conclusion:**

Incorporating the eGFR-cystatin C slope analysis supports the robustness of the previous creatinine-based slope findings, suggesting that the eGFR-creatinine slope analysis retains reasonable clinical reliability in patients treated with sodium-glucose cotransporter 2 inhibitors (SGLT2is).

## Graphical abstract

**Figure:**
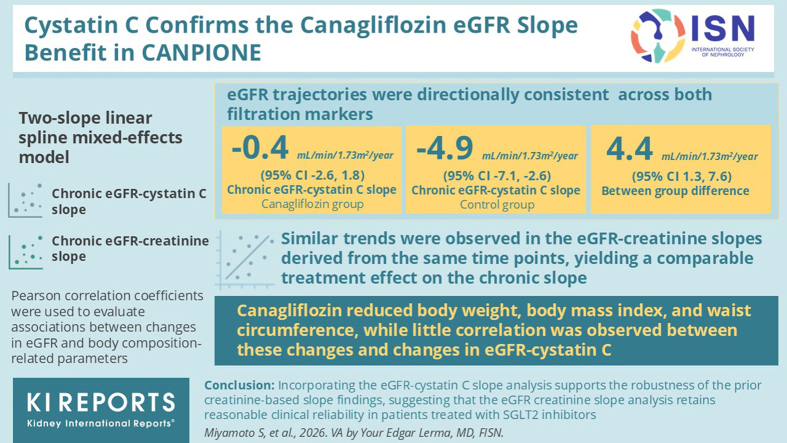

The CANPIONE study was a randomized, open-label, controlled trial designed to assess the kidney-protective effects of the SGLT2i canagliflozin, in Japanese patients with type 2 diabetes mellitus and microalbuminuria.^1^ A unique feature of this trial was the estimation of each participant’s change in eGFR slope by incorporating a 180-week preintervention eGFR slope before randomization. This allowed for a precise estimation of the treatment effect of canagliflozin, compared with usual care, on the natural course of eGFR decline at the individual level.

Our previous analysis demonstrated that canagliflozin attenuated kidney function decline, as evidenced by a significant between-group difference in the change in eGFR slope between the preintervention eGFR slope and the on-treatment chronic eGFR slope, in addition to a clinically meaningful reduction in albuminuria.^2^ These effects on the participant-specific trajectory of eGFR decline were primarily driven by improvements in the chronic slope. Alongside the improvement in eGFR trajectory, canagliflozin also induced a sustained reduction in BMI during chronic treatment.^2^

Serum creatinine concentration is determined not only by glomerular filtration but also by its generation in muscle tissues and dietary intake.^3^, ^4^, ^5^ Although previous body composition studies without a control group have suggested that weight loss associated with SGLT2is primarily because of reduction in fat mass, with minimal loss of lean or muscle mass,^6^^,^^7^ a recent meta-analysis of 25 randomized controlled trials revealed significant decreases in skeletal muscle mass with SGLT2is.^8^

In the CANPIONE study, the enrolled population had early-stage chronic kidney disease and relatively preserved kidney function, and the annual rate of eGFR decline before intervention was generally slow.^2^ Therefore, it is plausible that minor reductions in muscle mass during treatment might artifactually elevate serum creatinine-derived eGFR (eGFR-creatinine), thereby biasing the estimation of chronic eGFR-creatinine slopes.^9^^,^^10^ Cystatin C is an alternative endogenous filtration marker, which does not depend on muscle mass.^11^ Cystatin C-based eGFR can be used to determine if treatment induced changes in muscle mass may impact the observed benefits of SGLT2is on eGFR decline when estimated from creatinine.

In this prespecified exploratory analysis, we aimed to validate the robustness of the previously reported beneficial effect of canagliflozin on the eGFR-creatinine slope by using an eGFR-cystatin C slope during the chronic treatment period (weeks 4–52). Consequently, this analysis provides the first supportive evidence for the novel CANPIONE trial design, which incorporates the within-individual change in eGFR-creatinine slope from the preintervention to the chronic treatment period as a coprimary outcome measure.

## Methods

### Study Design, Participants, and Randomization

The CANPIONE study was a randomized, open-label, and parallel-group trial, conducted in 21 sites in Japan.^1^ The study was approved by the Okayama University certified review board and conducted in accordance with the Declaration of Helsinki and the Clinical Trials Act in Japan. The details of the eligibility criteria were described previously.^1^ In brief, participants were eligible if they had type 2 diabetes mellitus with microalbuminuria (first-morning geometric mean urine albumin-to-creatinine ratio of 50 to < 300 mg/g, confirmed at 2 separate study visits), an eGFR-creatinine of ≥ 45 ml/min per 1.73 m^2^, and glycated hemoglobin (HbA1c) of 6.5 to < 11.0% (48 to < 97 mmol/mol). A total of 98 eligible participants were randomized 1:1 to receive canagliflozin 100 mg once daily (canagliflozin group; *n* = 50) or to receive guideline-recommended standard care excluding the use of SGLT2is (control group; *n* = 48).^1^

### Procedure and Outcomes

During a 24-week preintervention period, participants were assessed for eligibility and randomized, and then entered a 52-week intervention period. At the end of the intervention period, participants assigned to the canagliflozin group discontinued canagliflozin treatment and entered a 4-week washout period during which background glucose-lowering medications remained unchanged.^1^

Blood samples were collected at all study visits and analyzed centrally. Serum cystatin C was measured at baseline and at weeks 4, 12, 28, 44, 52 (end of intervention), and 56 (end of washout). Serum creatinine and HbA1c were measured at all study visits as follows: every 8 weeks during the preintervention period (weeks −24, −16, and −8); at baseline, week 4, and thereafter every 8 weeks during the intervention period; and at week 56. Waist circumference was measured during the intervention period (at baseline, weeks 4, 28, and 52), and at the end of the washout period (week 56). Additionally, eGFR-creatinine values recorded in participants’ medical records at each study site from 3 years before the first study visit through the last study visit (week −180 to week 56), were also collected. eGFR-creatinine and eGFR-cystatin C were calculated using the Japanese equation for eGFR as described previously.^12^^,^^13^ The creatinine muscle index (CMI), an indicator of muscle mass, was calculated using the following formula^14^: CMI (mg/day/1.73 m^2^) = eGFR-cystatin C (ml/min/1.73 m^2^) × serum creatinine (mg/dl) × 1 dl/100 ml × 1440 min/day.

The main outcome of this prespecified exploratory analysis was the effect of canagliflozin versus control on the chronic eGFR-cystatin C slope (weeks 4–52).

### Statistical Analyses

Analyses were performed using the full analysis set, which included 96 randomized participants (49 and 47 for the canagliflozin and control groups, respectively) who received at least 1 assigned study treatment and had at least 1 postbaseline efficacy assessment.^2^ The effect of canagliflozin on eGFR-cystatin C and eGFR-creatinine slopes was estimated using a 2-slope linear spline mixed-effects model (with a knot at week 4), with unstructured covariance for random intercepts and slopes per participant. Least squares means were used to estimate 2 phases of eGFR slopes (acute slope: weeks 0–4 and chronic slope: weeks 4–52) for each treatment group, between-group differences at each phase, and their 95% CIs.

The subgroup analyses were performed using the same model, without adjustment for multiple comparisons, for the following variables: sex, age (< 65 vs. ≥ 65 years), duration of diabetes (< median vs. ≥ median years), baseline HbA1c (< 8% vs. ≥ 8%), baseline BMI (< 25 vs. ≥ 25 kg/m^2^), baseline systolic blood pressure (< 130 vs. ≥ 130 mmHg), baseline diastolic blood pressure (< 80 vs. ≥ 80 mmHg), baseline urine albumin-to-creatinine ratio (< 100 vs. ≥ 100 mg/g), 180-week preintervention eGFR-creatinine slope (subgroup 1: < −5, ≥ −5 to < −1, and ≥ −1 ml/min per 1.73 m^2^ per year; subgroup 2: < median vs. ≥ median ml/min per 1.73 m^2^ per year), baseline eGFR-creatinine and eGFR-cystatin C (< 60 vs. ≥ 60 ml/min per 1.73 m^2^), changes in eGFR-creatinine and eGFR-cystatin C from baseline to week 4 (< median vs. ≥ median in the canagliflozin group), changes in eGFR-creatinine and eGFR-cystatin C from weeks 52 to 56 (< median vs. ≥ median of the canagliflozin group), and the baseline use of angiotensin-converting enzyme inhibitors or angiotensin receptor blockers (yes vs. no).

For the analysis of the changes in body composition-related parameters, a mixed model for repeated measurements was used to estimate between-group differences. The dependent variable was the change in body weight, BMI, waist circumference, or CMI from baseline to weeks 4 to 52, and the independent variables included “treatment group,” “visit,” and “treatment group × visit” as fixed effects. The fixed effects were treated as categorical variables. An unstructured variance-covariance structure was assumed for the within-subject errors. The Kenward-Roger method was used to estimate the degree of freedom of distribution, and restricted maximum likelihood was used for estimation. Using the model, the least squares means and 2-sided CIs were estimated for each visit and for the between-group difference.

Pearson correlation coefficients were used to assess linear associations between the following: (i) eGFR-creatinine and eGFR-cystatin C values, (ii) changes in eGFR-creatinine and changes in eGFR-cystatin C, (iii) individual chronic eGFR-creatinine and eGFR-cystatin C slopes, and (iv) changes in eGFR and changes in body composition-related parameters (body weight, BMI, and waist circumference) or HbA1c.

*P*-values < 0.05 (2-sided) were considered significant. All statistical analyses were performed using R (version 4.5.2,The R Foundation, Vienna, Austria) or SAS (version 9.4,SAS Institute Inc., Cary, NC).

## Results

### Baseline Characteristics

Baseline characteristics were well balanced between the randomized groups (Table 1). Among the 96 participants, the mean eGFR-creatinine was 72.8 ml/min per 1.73 m^2^ (SD: 15.6)^2^ and the mean eGFR-cystatin C was 74.4 ml/min per 1.73 m^2^ (SD: 16.2). Overall, 22 and 20 participants (22.9% and 20.8%, respectively) had eGFR < 60 ml/min per 1.73 m^2^, when estimated using serum creatinine and cystatin C, respectively, whereas no participant had eGFR < 30 ml/min per 1.73 m^2^ at baseline. All participants had microalbuminuria at baseline with a median urine albumin-to-creatinine ratio of 104 mg/g (interquartile range: 80.2–150.4).^2^ Mean baseline body weight was similar between the canagliflozin and control groups (76.0 kg [SD: 14.0] and 75.4 kg [SD: 12.9], respectively).

**Table 1:** Baseline characteristics

	Canagliflozin (*n* = 49)	Control (*n* = 47)
Age, yr	61.3 (9.5)	61.2 (11.1)
Sex, male, *n* (%)	37 (75.5%)	34 (72.3%)
Duration of diabetes, yr^a^	14.9 (8.8)	15.2 (9.6)
Smoking status, yes, *n* (%)	18 (36.7%)	17 (36.2%)
Heart failure, *n* (%)	0 (0.0%)	0 (0.0%)
Myocardial infarction, *n* (%)	0 (0.0%)	0 (0.0%)
Cerebral infarction, *n* (%)	1 (2.0%)	2 (4.3%)
Renin angiotensin aldosterone system inhibitor use, *n* (%)	29 (59.2%)	33 (70.2%)
Diuretics use, *n* (%)	3 (6.1%)	2 (4.3%)
GLP-1 receptor agonists use, *n* (%)	8 (16.3%)	9 (19.1%)
Weight, kg	76.0 (14.0)	75.4 (12.9)
Body mass index, kg/m^2^	27.7 (4.6)	27.6 (4.4)
Waist circumference, cm^b^	96.1 (11.5)	95.9 (9.1)
Glycated hemoglobin, %	8.0 (1.2)	7.8 (1.1)
Systolic blood pressure, mm Hg	136.6 (18.3)	136.2 (14.9)
Geometric mean UACR, mg/g^c^	112.3 (87.7–167.7)	101.8 (73.2–136.6)
eGFR-creatinine, ml/min per 1.73 m^2^^d^	74.4 (15.2)	71.0 (15.9)
eGFR-creatinine ranges, ml/min per 1.73 m^2^		
< 60	12 (24.5%)	10 (21.3%)
≥ 60 to < 90	28 (57.1%)	29 (61.7%)
≥ 90^d^	9 (18.4%)	7 (14.9%)
eGFR-cystatin C, ml/min per 1.73 m^2^^d^	74.7 (15.2)	74.1 (17.4)
eGFR-cystatin C ranges, ml/min per 1.73 m^2^		
< 60	10 (20.4%)	10 (21.3%)
≥ 60 to < 90	30 (61.2%)	27 (57.4%)
≥90^d^	9 (18.4%)	9 (19.1%)

eGFR, estimated glomerular filtration rate; GLP-1, glucagon-like peptide-1; UACR, urine albumin-to-creatinine ratio.

Data are *n* (%), mean (SD), or median (IQR).

a Duration of diabetes mellitus was missing for 6 and 9 participants assigned to the canagliflozin and control group, respectively.

b Waist circumference was missing for 1 participant assigned to the canagliflozin group.

c Geometric mean of UACR values at 3 visits (weeks -16, -8, and 0).

d One participant assigned to the control group who had both creatinine- and cystatin C–based eGFR values > 140 ml/min per 1.73 m^2^ was excluded based on the predetermined Data Handling Rules for Statistical Analysis.

### Correlations Between Creatinine- and Cystatin C–based eGFR Values and Slopes

Baseline (*r* = 0.69), 52-week (*r* = 0.68), and 52-week change from baseline (*r* = 0.51) values correlated significantly between eGFR-creatinine and eGFR-cystatin C (Supplementary Figure S1A–C).

**Figure 1:**
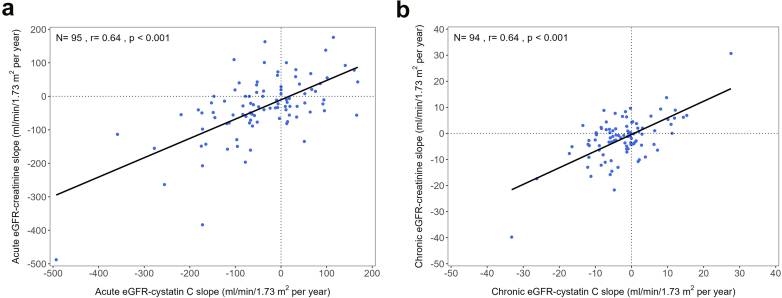
Correlations (Pearson’s *r*) between eGFR-cystatin C and eGFR-creatinine slopes at the individual participant level. Correlations between acute eGFR-cystatin C and acute eGFR-creatinine slopes (a) and chronic eGFR-cystatin C and chronic eGFR-creatinine slopes (b). One participant assigned to the canagliflozin group was excluded from the chronic slope analysis because eGFR values were missing after week 4 during the intervention period. One participant assigned to the control group who had both creatinine- and cystatin C–based eGFR values >140 ml/min per 1.73 m^2^ during the intervention period was excluded based on the predetermined data handling rules for statistical analysis. eGFR, estimated glomerular filtration rate

At the individual participant level, acute and chronic eGFR-creatinine slopes were moderately correlated with acute (*r* = 0.64) and chronic (*r* = 0.64) eGFR-cystatin C slopes, respectively (Figure 1a and b), suggesting the consistency of kidney function trajectories across the 2 filtration markers.

### Effect of Canagliflozin on the eGFR-Cystatin C slope—Validation of the Robustness of eGFR-Creatinine-Based Findings

**Figure 2:**
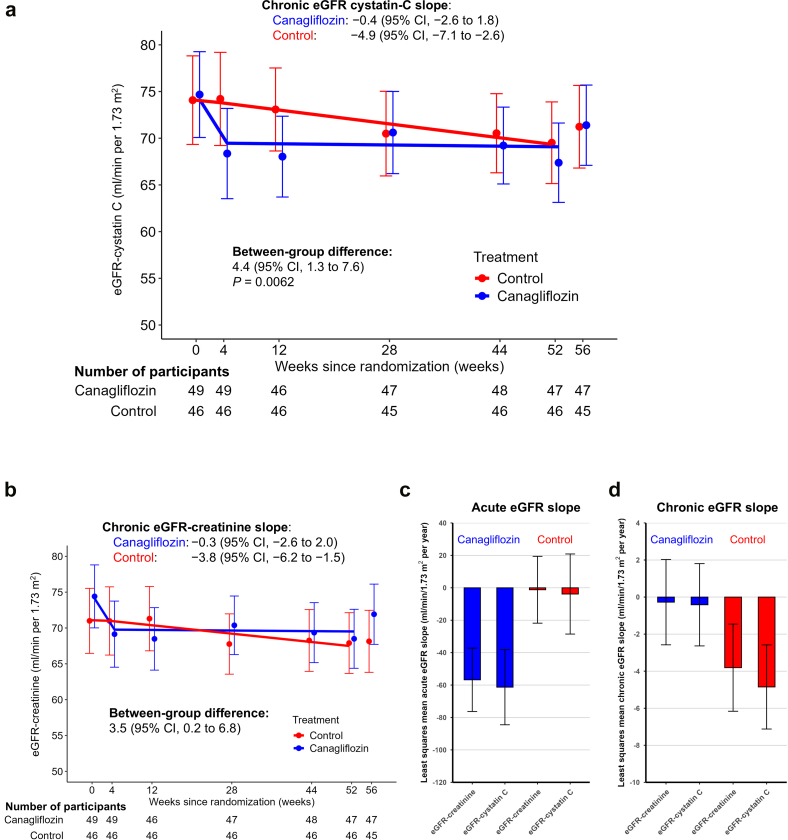
Least squares mean eGFR-cystatin C and eGFR-creatinine slopes over time. (a) Least squares mean eGFR-cystatin C slopes over time. (b) Least squares mean eGFR-creatinine slopes over time estimated based on the limited eGFR-creatinine data from the same time points (weeks 0, 4, 12, 28, 44, and 52) as eGFR-cystatin C measurements. (c) Least squares mean acute eGFR-creatinine and eGFR-cystatin C slopes. (d) Least squares mean chronic eGFR-creatinine and eGFR-cystatin C slopes. Error bars show 95% CIs. CI, confidence interval; eGFR, estimated glomerular filtration rate.

**Figure 3:**
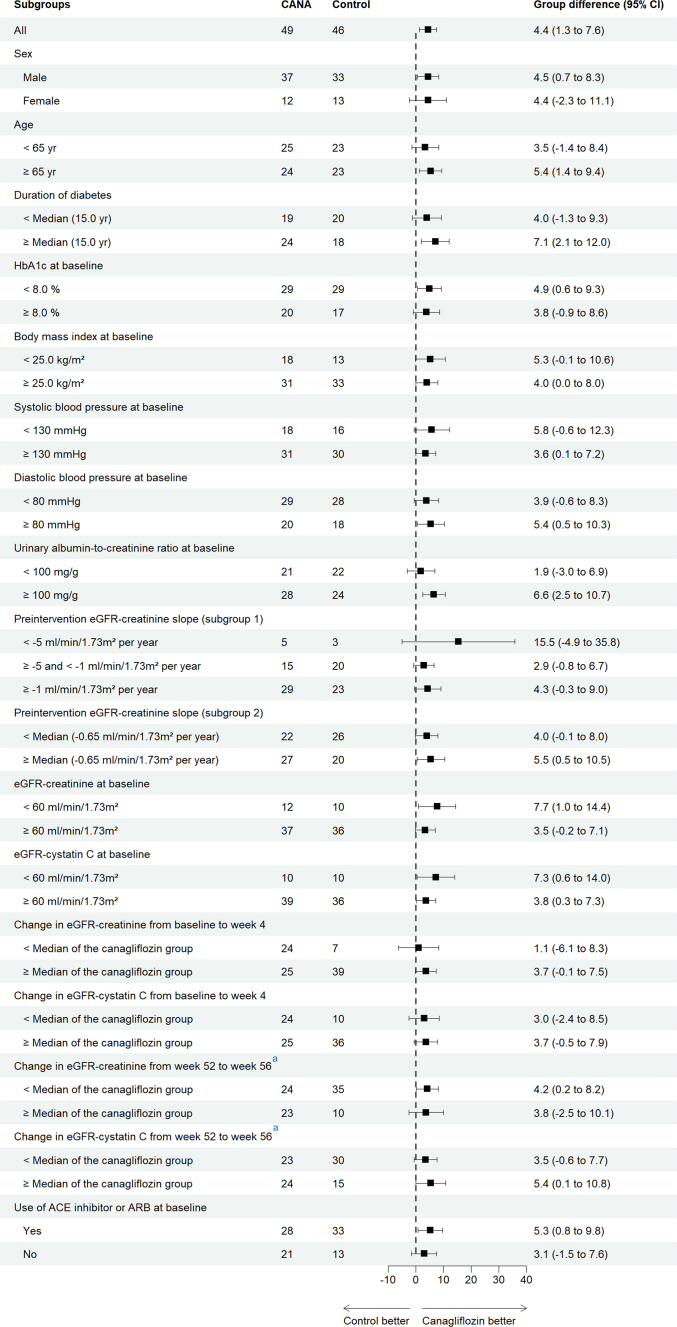
Differences in the effect of canagliflozin versus control on the chronic eGFR-cystatin C slope by prespecified subgroups. Error bars show 95% CIs. One participant assigned to the control group who had eGFR values over 140 ml/min per 1.73 m^2^ during the preintervention period and at baseline, weeks 4, 52, and 56 was excluded based on the predetermined data handling rules for statistical analysis. ^a^Two and 1 participants assigned to the canagliflozin and control groups, respectively, were excluded from the analysis because eGFR values were missing at either week 52 or 56, or both. ACE, angiotensin-converting enzyme; ARB, angiotensin receptor blocker; CI, confidence interval; eGFR, estimated glomerular filtration rate; HbA1c, glycated hemoglobin.

When estimated using eGFR-cystatin C, the eGFR-cystatin C slopes in the canagliflozin group exhibited a biphasic pattern characterized by an acute decline during weeks 0 to 4 followed by stabilization during the chronic treatment period (weeks 4–52). The least-squares mean on-treatment (chronic) eGFR-cystatin C slope, the main outcome of this prespecified analysis, showed a significantly slower decline in the canagliflozin group compared with the control group as follows: −0.4 (95% CI: −2.6 to 1.8) and −4.9 (95% CI: −7.1 to −2.6) ml/min per 1.73 m^2^ per year for the canagliflozin and control groups, respectively, corresponding to a between-group difference of 4.4 ml/min per 1.73 m^2^ per year (95% CI: 1.3–7.6, *P* = 0.0062) (Figure 2a and Supplementary Table S1). Subgroup analyses indicated that the treatment effects of canagliflozin on the chronic eGFR-cystatin C slope were consistent across prespecified subgroups (Figure 3 and Supplementary Table S2).

To further validate the robustness of the creatinine-based findings, eGFR-creatinine slopes were estimated using eGFR-creatinine values from the same time points (weeks 0, 4, 12, 28, 44, and 52) as the eGFR-cystatin C measurements. In the canagliflozin group, a similar biphasic pattern was observed for the eGFR-creatinine slopes, resulting in a comparable treatment effect, with a between-group difference of 3.5 ml/min per 1.73 m^2^ per year (95% CI: 0.2–6.8) (Figure 2b–d and Supplementary Table S3).

**Figure 4:**
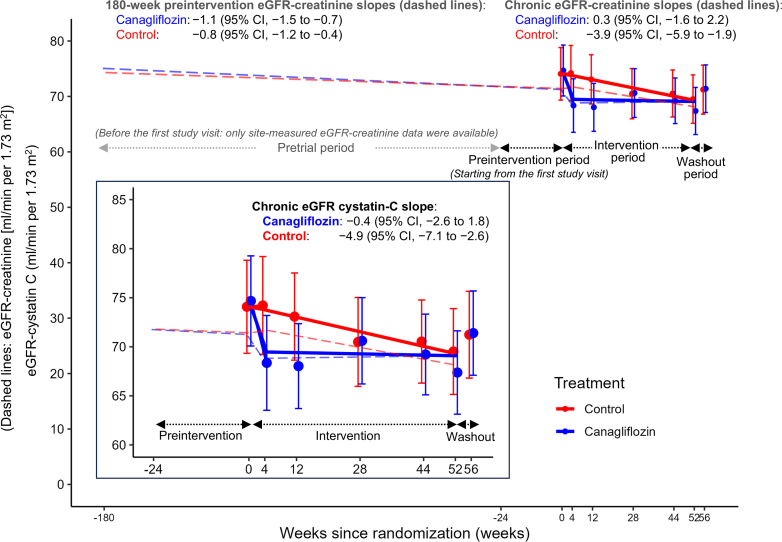
Least squares mean eGFR-cystatin C slopes superimposed on the change in eGFR-creatinine slopes established using both centrally- and site-measured eGFR data (dashed lines). Error bars show 95% CIs. CI, confidence interval; eGFR, estimated glomerular filtration rate.

In our previous report of the CANPIONE study, we assessed the effect of canagliflozin on kidney function decline as the second coprimary outcome, by evaluating the change in eGFR slopes between the 180-week preintervention eGFR slope (eGFR slope before intervention) and on-treatment eGFR slope using both centrally- and site-measured eGFR data.^2^ The chronic eGFR-creatinine slopes estimated using both centrally- and site-measured eGFR data were 0.3 (95% CI: −1.6 to 2.2) and −3.9 (95% CI: −5.9 to −1.9) ml/min per 1.73 m^2^ per year in the canagliflozin and control groups, respectively, corresponding to a between-group difference of 4.2 (95% CI: 1.4–6.9) ml/min per 1.73 m^2^ per year,^2^ which was also similar to the chronic eGFR-cystatin C slopes in the current study. Figure 4 shows the least squares mean eGFR-cystatin C slopes superimposed on the change in eGFR-creatinine slopes (dashed lines) established using both centrally- and site-measured eGFR data in the CANPIONE study. The robust treatment effect of canagliflozin on the rate of eGFR-creatinine decline was also evident when assessed using the chronic eGFR-cystatin C slope.

### Associations Between eGFR and Parameters Related to Body Composition

Little correlation was observed between baseline eGFR-cystatin C and baseline body weight, BMI, waist circumference, or HbA1c (Supplementary Figure S2A, C, E, and G). Similarly, baseline eGFR-creatinine did not correlate with baseline body weight, BMI, or waist circumference but a weak correlation was observed between baseline eGFR-creatinine and baseline HbA1c (Supplementary Figure S2B, D, F, and H).

Least squares mean changes from baseline in body weight, BMI,^2^ and waist circumference at week 52 are summarized in Supplementary Figure S3A to C. Canagliflozin treatment, as compared with usual care, showed robust reductions in least squares mean body weight (−3.0 kg [95% CI: −4.1 to −1.9]), BMI (−1.1 kg/m^2^ [95% CI: −1.5 to −0.7]) and waist circumference (−2.5 cm [95% CI: −4.3 to −0.7]). Similar to the previously reported effect on BMI,^2^ the treatment effects of canagliflozin on body weight and waist circumference were largely maintained during the intervention period (Supplementary Figure S4A and B).

To assess whether the observed reductions in these parameters had any impact on the trajectories of eGFR-cystatin C and eGFR-creatinine, we examined their correlations with changes in eGFR-cystatin C or eGFR-creatinine. When assessed using pooled data from both groups, changes from baseline in body weight, BMI, waist circumference, or HbA1c at week 52 did not correlate with changes from baseline in eGFR-creatinine or eGFR-cystatin C at week 52 (Supplementary Figure S5A–H). Similarly, in the canagliflozin group, changes from baseline in body weight, BMI, waist circumference, or HbA1c at week 52 did not correlate with changes in eGFR-creatinine or eGFR-cystatin C (Supplementary Figure S6A–H).

### Distribution of the Creatinine Muscle Index

We further examined if the CMI, defined as an index of muscle mass based on creatinine filtration, was affected by the canagliflozin treatment. The distribution of baseline CMI and the change from baseline in CMI at week 52 was comparable between groups (Supplementary Figure S7A and B). Additionally, there was no difference in least squares mean change from baseline at week 52 in CMI (Supplementary Figure S8).

## Discussion

In these prespecified analyses from the CANPIONE study, canagliflozin, compared with usual care, showed a robust and statistically significant effect in slowing the rate of eGFR-cystatin C decline during chronic treatment. Notably, the effect size for the chronic eGFR-cystatin C slope was comparable to that of the previously reported chronic eGFR-creatinine slope—which was estimated more precisely using both centrally- and site-measured data and by incorporating within-individual changes in eGFR slope^2^—supporting a robust effect of canagliflozin in reducing eGFR decline. Although canagliflozin reduced body weight and BMI, neither of these changes was significantly correlated with changes in eGFR-creatinine or eGFR-cystatin C, suggesting that the previously observed benefit of canagliflozin on the eGFR-creatinine slope was unlikely biased by the effect of canagliflozin on body weight or muscle mass. In fact, CMI, a marker for muscle mass,^14^ did not show apparent change after canagliflozin treatment as compared with the control group, supporting the notion that canagliflozin did not change muscle mass.

Although acute changes in eGFR following the initiation of SGLT2is are primarily attributed to hemodynamic effects on single-nephron GFR, changes in the chronic eGFR slope are generally assumed to reflect reductions in functioning nephron loss and are therefore considered to confer long-term kidney protection.^5^^,^^15^, ^16^, ^17^ Thus, our findings support the notion that the observed treatment effect in slowing the rate of eGFR-cystatin C or eGFR-creatinine decline with canagliflozin may reflect actual preservation of kidney function.

In clinical trials targeting early-stage chronic kidney disease, detecting meaningful treatment effects using the eGFR slope, a validated surrogate end point for kidney failure, ideally requires 3 years of follow-up with a substantial number of participants.^18^^,^^19^ To facilitate clinical trials for kidney protection at an early stage of chronic kidney disease, we proposed a trial design that incorporated each participant’s preintervention eGFR slope to assess treatment effects on the natural course of kidney function decline.^1^ In the CANPIONE study with this novel design feature, canagliflozin compared with usual care significantly reduced the participant’s individual change in eGFR slope (i.e., slowed the rate of kidney function decline), in addition to the clinically meaningful 30.8% relative reduction in albuminuria.^2^ This approach enabled sensitive detection of treatment effects, and in fact, the benefit of canagliflozin in slowing eGFR decline was established with 1 year of follow-up in fewer than 100 participants at relatively low risk for kidney disease progression.^2^^,^^20^ Notably, current prespecified analyses using eGFR-cystatin C complement our previous findings based on eGFR-creatinine and add strength by demonstrating that a filtration marker unaffected by muscle mass yielded consistent results. Together, these findings further support the beneficial effect of canagliflozin on kidney protection and, importantly, provide the first supportive evidence for the validity of the novel CANPIONE trial design that evaluates the within-individual change in eGFR-creatinine slope as a coprimary outcome measure.

Our study has several limitations. First, for estimating the chronic slope, the number of eGFR-cystatin C measurements was relatively small—5 measurements per participant—and the duration was limited to 48 weeks, which may have affected the precision of slope estimates. However, the width of 95% CIs for the chronic eGFR-cystatin C was comparable to that of the eGFR-creatinine slope, which was estimated using 7 centrally-measured eGFR values and additional site-measured eGFR data. Second, eGFR-cystatin C was not measured before randomization, and therefore, we were unable to estimate the rate of eGFR-cystatin C decline before randomization. Third, the open-label nature of the CANPIONE study may have introduced potential bias in the assessment of study outcomes. Fourth, the relatively small sample size limits generalizability of the findings; therefore, larger prospective clinical trials are required to confirm these results. Nevertheless, despite the limited sample size, our study demonstrated robust and statistically significant differences in eGFR-cystatin C slope, as well as previously reported significant effects on both primary outcomes of the main trial.^2^ Finally, this study was conducted during the COVID-19 pandemic, which necessitated a preplanned sample size re-estimation and the modification of primary outcomes^2^—factors that may have affected the overall study findings.

In conclusion, canagliflozin slowed the rate of chronic kidney function decline in participants with type 2 diabetes mellitus and microalbuminuria, as assessed using both creatinine- and cystatin C-based eGFR slopes. These effects were independent of changes in body composition-related parameters and support the robust kidney-protective effects of canagliflozin in early-stage diabetic kidney disease. In addition to the within-individual change in eGFR-creatinine slope, incorporating the eGFR-cystatin C slope as an additive outcome measure may represent a novel approach to determine and further reinforce the kidney protective potential of new therapies in early-stage chronic kidney disease.

## Disclosure

SM reports speaker honoraria from Daiichi Sankyo, Mitsubishi Tanabe, and Bayer. HJLH has served as a consultant for AstraZeneca, Alexion, Amgen, Alnylam, Bayer, Boehringer Ingelheim, Biocity Biopharmaceuticals, DImerix, Eli Lilly, Novo Nordisk, Novartis, Roche, and Travere Therapeutics. He also reports research funding from AstraZeneca, Boehringer Ingelheim, Bayer, Novo Nordisk, and Janssen and honoraria (speakers bureau) from AstraZeneca and Novo Nordisk. MY has received stock dividends from Takeda. MT reports speaker honoraria from Taisho, Kissei, Medtronic, Terumo, Abbott, MSD, Eli Lilly, Novartis, Takeda, Sumitomo, Sanofi, Novo Nordisk, Daiichi Sankyo, Mitsubishi Tanabe, Ono, Boehringer Ingelheim, AstraZeneca, Astellas, Kyowa Kirin, and Kowa and research grants from LifeScan Japan, Mitsubishi Tanabe, Daiichi Sankyo, Novo Nordisk, Sanofi, Takeda, Eli Lilly, MSD, Roche, and Dexcom. DS reports speaker honoraria for speaking from MSD, Eli Lilly Japan, and Nippon Boehringer Ingelheim. HA reports speaker honoraria from Arkray, Sumitomo Pharma, Kowa, Kyowa Hakko Kirin, Taisho, Novo Nordisk, Mitsubishi Tanabe, Daiichi Sankyo, Novartis, Sanwa Chemistry, Kissei, Mochida, Bayer, Teijin, and Sawai. MK belongs to the endowed department sponsored by Boehringer Ingelheim, Mitsubishi Tanabe, Ono Pharmaceuticals, and Taisho Toyama Pharmaceuticals; and reports research grants from Mitsubishi Tanabe, AstraZeneca, Sanwa Kagaku Kenkyusho, and Kyowa Kirin. AN reports speaker honoraria from Mitsubishi Tanabe, Fukuda Denshi, Astellas, Chugai, Novartis, MSD, Takeda, Kissei, Kyowa Hakko Kirin, Sanofi, Bristol-Myers, Kowa, Sanwa Kagaku Kenkyusho, and Ono Pharmaceuticals. TN reports speaker honoraria from Eli Lilly, Novo Nordisk, and Sumitomo Dainippon. KF reports speaker honoraria from Sanofi, Merck Sharpe & Dohme, Taisho, Eli Lilly, Terumo, Arkray, Astellas, AstraZeneca, Boehringer Ingelheim, Mitsubishi Tanabe, Ono Pharmaceuticals, Novo Nordisk, Kissei, Sumitomo Dainippon, Kowa, Takeda, Daiichi Sankyo, Chugai, Abbott, Otsuka, and Kyowa Hakko Kirin. DK reports speaker honoraria from Novo Nordisk, Sanofi, Boehringer Ingelheim, Kyowa Kirin, Sumitomo Pharma, Ono Pharmaceuticals, Kowa, Daiichi Sankyo, MSD, Eli Lilly, and Mitsubishi Tanabe; and research grants from Mitsubishi Tanabe, Nipro, Eli Lilly, Böehringer Ingelheim, and Sumitomo Pharma. NM reports speaker honorarium from Kowa and Novo Nordisk. KSh reports speaker honoraria for speaking from MSD, Eli Lilly, Boehringer Ingelheim, Novo Nordisk, Mitsubishi Tanabe, and Kyowa Hakko Kirin; research supports from Takeda, MSD, Kyowa Hakko Kirin, and Mitsubishi Tanabe; and a consulting fee from Daiichi Sankyo. All other authors have no conflict of interest to disclose.

## Patient Consent

All participants provided written informed consent. This study was registered with the University Hospital Medical Information Network (UMIN000029905) and the Japan Registry of Clinical Trials (jRCTs061180047), in accordance with the Clinical Trials Act.

## Supplementary Material

Supplementary File (PDF) Figure S1. Correlations (Pearson’s *r*) between eGFR-cystatin C and eGFR-creatinine. Figure S2. Correlations (Pearson’s *r*) between eGFR (cystatin C and creatinine) and body weight, BMI, waist circumference, and HbA1c. Figure S3. Least squares mean changes from baseline in parameters related to body composition at week 52. Figure S4. Least squares mean changes from baseline in parameters related to body composition over time. Figure S5. Correlations (Pearson’s *r*) between changes from baseline in eGFR (cystatin C and creatinine) and in body weight, BMI, waist circumference, and HbA1c at week 52. Figure S6. Correlations (Pearson’s *r*) between changes from baseline in eGFR (cystatin C and creatinine) and body weight, BMI, waist circumference, and HbA1c at week 52 in the canagliflozin group. Figure S7. Comparison of the distribution of creatinine muscle index (CMI) in the canagliflozin and control groups. Figure S8. Least squares mean change from baseline in CMI at week 52. Table S1. The main outcome of this prespecified exploratory analysis and related least squares mean eGFR-cystatin C slopes based on a 2-slope linear spline mixed effects model. Table S2. Prespecified subgroup analyses of the main outcome and related least-squares mean eGFR-cystatin C slopes. Table S3. Least squares mean eGFR-creatinine slopes based on a 2-slope linear spline mixed effects model using data from the same time points (weeks 0, 4, 12, 28, 44, and 52) as eGFR-cystatin C measurement. CONSORT Checklist.
