# Supplementary material for: Cystatin C Confirms the Canagliflozin eGFR Slope Benefit in CANPIONE
Source: Kidney Int Rep. 2026 Feb 23;11(5):106374. doi: 10.1016/j.ekir.2026.106374 (PMC13022597; doi:10.1016/j.ekir.2026.106374)
Supplement: Supplementary File (PDF) — Figure S1. Correlations (Pearson’s r) between eGFR-cystatin C and eGFR-creatinine. Figure S2. Correlations (Pearson’s r) between eGFR (cystatin C and creatinine) and body weight, BMI, waist circumference, and HbA1c. Figure S3. Least squares mean changes from baseline in parameters related to body composition at week 52. Figure S4. Least squares mean changes from baseline in parameters related to body composition over time. Figure S5. Correlations (Pearson’s r) between changes from baseline in eGFR (cystatin C and creatinine) and in body weight, BMI, waist circumference, and HbA1c at week 52. Figure S6. Correlations (Pearson’s r) between changes from baseline in eGFR (cystatin C and creatinine) and body weight, BMI, waist circumference, and HbA1c at week 52 in the canagliflozin group. Figure S7. Comparison of the distribution of creatinine muscle index (CMI) in the canagliflozin and control groups. Figure S8. Least squares mean change from baseline in CMI at week 52. Table S1. The main outcome of this prespecified exploratory analysis and related least squares mean eGFR-cystatin C slopes based on a 2-slope linear spline mixed effects model. Table S2. Prespecified subgroup analyses of the main outcome and related least-squares mean eGFR-cystatin C slopes. Table S3. Least squares mean eGFR-creatinine slopes based on a 2-slope linear spline mixed effects model using data from the same time points (weeks 0, 4, 12, 28, 44, and 52) as eGFR-cystatin C measurement. CONSORT Checklist. [file mmc1.pdf]

Cystatin C Confirms the Canagliflozin eGFR Slope Benefit in CANPIONE

Supplementary Material

Table of Contents

Supplementary Tables

Supplemental Table S1: The main outcome of this prespecified exploratory analysis and related least squares mean eGFR-cystatin C slopes based on a two-slope linear spline mixed effects model. 1

Supplemental Table S2: Prespecified subgroup analyses of the main outcome and related least-squares mean eGFR-cystatin C slopes. 2

Supplemental Table S3: Least squares mean eGFR-creatinine slopes based on a two-slope linear spline mixed effects model using data from the same time points (weeks 0, 4, 12, 28, 44, and 52) as eGFR-cystatin C measurement. 12

Supplementary Figures

Supplemental Figure S1: Correlations (Pearson's r) between eGFR-cystatin C and eGFR-creatinine. 13

Supplemental Figure S2: Correlations (Pearson's r) between eGFR (cystatin C and creatinine) and body weight, BMI, waist circumference, and HbA1c. 14

Supplemental Figure S3: Least squares mean changes from baseline in parameters related to body composition at week 52. 16

Supplemental Figure S4: Least squares mean changes from baseline in parameters related to body composition over time. 17

Supplemental Figure S5: Correlations (Pearson's r) between changes from baseline in eGFR (cystatin C and creatinine) and body weight, BMI, waist circumference, and HbA1c at week 52. 18

Supplemental Figure S6: Correlations (Pearson's r) between changes from baseline in eGFR (cystatin C and creatinine) and body weight, BMI, waist circumference, and HbA1c at week 52 in the canagliflozin group. 20

Supplemental Figure S7: Comparison of the distribution of creatinine muscle index (CMI) in the canagliflozin and control groups. 22

Supplemental Figure S8: Least squares mean change from baseline in CMI at week 52. 23

The CONSORT reporting checklist 24

Supplementary Tables

Supplementary Table S1: The main outcome of this prespecified exploratory analysis and related least squares mean eGFR-cystatin C slopes based on a two-slope linear spline mixed effects model.

eGFR-cystatin C slopes	Estimate (ml/min/1.73m^2 per year)	*P*	95% CI Lower	95% CI Upper
Acute slope for Canagliflozin	−61.3	-	−84.5	−38.1
Acute slope for Control	−3.8	-	−28.5	20.9
Chronic slope for Canagliflozin	−0.4	-	−2.6	1.8
Chronic slope for Control	−4.9	-	−7.1	−2.6
Difference of acute slope (Canagliflozin − Control)	−57.5	-	−91.3	−23.6
Difference of chronic slope (Canagliflozin − Control)	4.4	0.0062	1.3	7.6

eGFR, estimated glomerular filtration rate.

Supplementary Table S2: Prespecified subgroup analyses of the main outcome and related least-squares mean eGFR-cystatin C slopes

Subgroup	Category	eGFR-cystatin C slope	Estimate (ml/min/1.73m^2 per year)	95% CI Lower	95% CI Upper
Sex	Male (n=70)	Acute slope for Canagliflozin	−60.9	−89.7	−32.1
	(Canagliflozin: n=37, Control: n=33)	Acute slope for Control	−3.3	−35.5	29.0
		Chronic slope for Canagliflozin	−0.4	−3.1	2.2
		Chronic slope for Control	−4.9	−7.7	−2.2
		Difference of acute slope	−57.7	−100.9	−14.5
		Difference of chronic slope	4.5	0.7	8.3
	Female (n=25)	Acute slope for Canagliflozin	−68.8	−116.8	−20.9
	(Canagliflozin: n=12, Control: n=13)	Acute slope for Control	−5.3	−47.0	36.3
		Chronic slope for Canagliflozin	−0.2	−5.1	4.7
		Chronic slope for Control	−4.6	−9.2	−0.1
		Difference of acute slope	−63.5	−127.0	0.0
		Difference of chronic slope	4.4	−2.3	11.1
Age	< 65 yr (n=48)	Acute slope for Canagliflozin	−53.3	−87.0	−19.6
	(Canagliflozin: n=25, Control: n=23)	Acute slope for Control	−18.3	−55.6	19.0
		Chronic slope for Canagliflozin	−2.2	−5.5	1.2
		Chronic slope for Control	−5.7	−9.2	−2.1
		Difference of acute slope	−35.0	−85.2	15.3
		Difference of chronic slope	3.5	−1.4	8.4
	≥ 65 yr (n=47)	Acute slope for Canagliflozin	−69.5	−102.2	−36.9

	(Canagliflozin: n=24, Control: n=23)	Acute slope for Control	8.6	−24.9	42.1
		Chronic slope for Canagliflozin	1.4	−1.5	4.3
		Chronic slope for Control	−4.0	−6.9	−1.2
		Difference of acute slope	−78.1	−125.0	−31.3
		Difference of chronic slope	5.4	1.4	9.4
Duration of diabetes	< Median (yr, n=39)	Acute slope for Canagliflozin	−65.2	−102.8	−27.6
(median= 15.0 yr)	(Canagliflozin: n=19, Control: n=20)	Acute slope for Control	−23.4	−60.1	13.4
		Chronic slope for Canagliflozin	−0.1	−3.9	3.6
		Chronic slope for Control	−4.1	−7.8	−0.5
		Difference of acute slope	−41.8	−94.4	10.8
		Difference of chronic slope	4.0	−1.3	9.3
	≥ Median (yr, n=42)	Acute slope for Canagliflozin	−75.4	−111.1	−39.8
	(Canagliflozin: n=24, Control: n=18)	Acute slope for Control	29.0	−12.3	70.3
		Chronic slope for Canagliflozin	0.5	−2.7	3.8
		Chronic slope for Control	−6.5	−10.3	−2.8
		Difference of acute slope	−104.4	−159.0	−49.8
		Difference of chronic slope	7.1	2.1	12.0
HbA1c at baseline	< 8.0 % (n=58)	Acute slope for Canagliflozin	−63.3	−95.9	−30.7
	(Canagliflozin: n=29, Control: n=29)	Acute slope for Control	−13.2	−46.2	19.7
		Chronic slope for Canagliflozin	0.3	−2.8	3.4
		Chronic slope for Control	−4.6	−7.7	−1.6
		Difference of acute slope	−50.1	−96.4	−3.8

		Difference of chronic slope	4.9	0.6	9.3
	≥ 8.0 % (n=37)	Acute slope for Canagliflozin	−63.2	−96.6	−29.9
	(Canagliflozin: n=20, Control: n=17)	Acute slope for Control	10.8	−27.4	48.9
		Chronic slope for Canagliflozin	−1.4	−4.6	1.9
		Chronic slope for Control	−5.2	−8.7	−1.8
		Difference of acute slope	−74.0	−124.7	−23.3
		Difference of chronic slope	3.8	−0.9	8.6
BMI at baseline	< 25.0 kg/m^2 (n=31)	Acute slope for Canagliflozin	−50.8	−87.4	−14.2
	(Canagliflozin: n=18, Control: n=13)	Acute slope for Control	−16.4	−59.7	26.9
		Chronic slope for Canagliflozin	1.1	−2.5	4.6
		Chronic slope for Control	−4.2	−8.2	−0.2
		Difference of acute slope	−34.4	−91.1	22.3
		Difference of chronic slope	5.3	−0.1	10.6
	≥ 25.0 kg/m^2 (n=64)	Acute slope for Canagliflozin	−67.3	−97.0	−37.6
	(Canagliflozin: n=31, Control: n=33)	Acute slope for Control	3.1	−26.8	33.0
		Chronic slope for Canagliflozin	−1.2	−4.0	1.7
		Chronic slope for Control	−5.2	−8.0	−2.4
		Difference of acute slope	−70.4	−112.6	−28.3
		Difference of chronic slope	4.0	0.0	8.0
Systolic blood pressure at baseline	< 130 mmHg (n=34)	Acute slope for Canagliflozin	−31.9	−73.2	9.5
	(Canagliflozin: n=18, Control: n=16)	Acute slope for Control	9.3	−37.6	56.2
		Chronic slope for Canagliflozin	1.3	−3.1	5.7

		Chronic slope for Control	−4.5	−9.2	0.2
		Difference of acute slope	−41.2	−103.7	21.3
		Difference of chronic slope	5.8	−0.6	12.3
	≥ 130 mmHg (n=61)	Acute slope for Canagliflozin	−79.6	−107.0	−52.2
	(Canagliflozin: n=31, Control: n=30)	Acute slope for Control	−11.0	−39.1	17.1
		Chronic slope for Canagliflozin	−1.4	−3.9	1.1
		Chronic slope for Control	−5.1	−7.6	−2.5
		Difference of acute slope	−68.6	−107.9	−29.3
		Difference of chronic slope	3.6	0.1	7.2
Diastolic blood pressure at baseline	< 80 mmHg (n=57)	Acute slope for Canagliflozin	−40.7	−74.4	−6.9
	(Canagliflozin: n=29, Control: n=28)	Acute slope for Control	7.5	−27.2	42.2
		Chronic slope for Canagliflozin	−0.6	−3.7	2.5
		Chronic slope for Control	−4.4	−7.6	−1.3
		Difference of acute slope	−48.2	−96.6	0.2
		Difference of chronic slope	3.9	−0.6	8.3
	≥ 80 mmHg (n=38)	Acute slope for Canagliflozin	−92.2	−124.2	−60.1
	(Canagliflozin: n=20, Control: n=18)	Acute slope for Control	−21.0	−56.5	14.6
		Chronic slope for Canagliflozin	−0.1	−3.5	3.3
		Chronic slope for Control	−5.5	−9.0	−2.0
		Difference of acute slope	−71.2	−119.1	−23.3
		Difference of chronic slope	5.4	0.5	10.3
UACR at baseline	< 100 mg/g (n=43)	Acute slope for Canagliflozin	−71.5	−110.7	−32.3

	(Canagliflozin: n=21, Control: n=22)	Acute slope for Control	4.1	−35.8	44.0
		Chronic slope for Canagliflozin	−0.8	−4.3	2.8
		Chronic slope for Control	−2.7	−6.1	0.7
		Difference of acute slope	−75.6	−131.5	−19.7
		Difference of chronic slope	1.9	−3.0	6.9
	≥ 100 mg/g (n=52)	Acute slope for Canagliflozin	−53.2	−82.0	−24.5
	(Canagliflozin: n=28, Control: n=24)	Acute slope for Control	−12.3	−44.2	19.6
		Chronic slope for Canagliflozin	−0.1	−2.9	2.7
		Chronic slope for Control	−6.7	−9.7	−3.7
		Difference of acute slope	−40.9	−83.9	2.0
		Difference of chronic slope	6.6	2.5	10.7
180-week preintervention	< −5 ml/min/1.73m^2 per year (n=8)	Acute slope for Canagliflozin	−114.3	−242.7	14.1
eGFR-creatinine slope[a]	(Canagliflozin: n=5, Control: n=3)	Acute slope for Control	3.2	−153.6	159.9
		Chronic slope for Canagliflozin	4.3	−8.3	16.9
		Chronic slope for Control	−11.1	−27.2	4.9
		Difference of acute slope	−117.4	−320.0	85.1
		Difference of chronic slope	15.5	−4.9	35.8
	≥ −5 and < −1 ml/min/1.73m^2 per year	Acute slope for Canagliflozin	−56.8	−99.8	−13.8
	(n=35)	Acute slope for Control	−25.5	−62.9	12.0
	(Canagliflozin: n=15, Control: n=20)	Chronic slope for Canagliflozin	0.0	−2.8	2.8
		Chronic slope for Control	−3.0	−5.4	−0.5
		Difference of acute slope	−31.4	−88.3	25.6

		Difference of chronic slope	2.9	−0.8	6.7
	≥ −1 ml/min/1.73 m^2 per year (n=52) (Canagliflozin: n=29, Control: n=23)	Acute slope for Canagliflozin	−58.6	−86.3	−30.9
		Acute slope for Control	14.2	−18.3	46.8
		Chronic slope for Canagliflozin	−1.3	−4.5	1.8
		Chronic slope for Control	−5.7	−9.2	−2.2
		Difference of acute slope	−72.8	−115.6	−30.1
		Difference of chronic slope	4.3	−0.3	9.0
180-week preintervention eGFR-creatinine slope[a] (Median= −0.650)	< Median (ml/min/1.73 m^2 per year, n=48) (Canagliflozin: n=22, Control: n=26)	Acute slope for Canagliflozin	−63.8	−103.4	−24.3
		Acute slope for Control	−20.0	−56.7	16.6
		Chronic slope for Canagliflozin	0.2	−2.7	3.2
		Chronic slope for Control	−3.7	−6.5	−1.0
		Difference of acute slope	−43.8	−97.7	10.1
		Difference of chronic slope	4.0	−0.1	8.0
	≥ Median (ml/min/1.73 m^2 per year, n=47) (Canagliflozin: n=27, Control: n=20)	Acute slope for Canagliflozin	−64.2	−92.8	−35.6
		Acute slope for Control	17.5	−16.8	51.9
		Chronic slope for Canagliflozin	−0.8	−4.1	2.5
		Chronic slope for Control	−6.3	−10.0	−2.5
		Difference of acute slope	−81.7	−126.4	−37.0
		Difference of chronic slope	5.5	0.5	10.5
eGFR-creatinine at baseline[a]	< 60 ml/min/1.73m^2 (n=22) (Canagliflozin: n=12, Control: n=10)	Acute slope for Canagliflozin	−84.1	−131.3	−37.0
		Acute slope for Control	29.8	−19.6	79.1
		Chronic slope for Canagliflozin	2.0	−2.5	6.6

		Chronic slope for Control	−5.7	−10.6	−0.8
		Difference of acute slope	−113.9	−182.2	−45.6
		Difference of chronic slope	7.7	1.0	14.4
	≥ 60 ml/min/1.73m^2 (n=73)	Acute slope for Canagliflozin	−55.4	−81.3	−29.5
	(Canagliflozin: n=37, Control: n=36)	Acute slope for Control	−12.3	−40.2	15.6
		Chronic slope for Canagliflozin	−1.1	−3.7	1.4
		Chronic slope for Control	−4.6	−7.2	−2.0
		Difference of acute slope	−43.1	−81.2	−5.1
		Difference of chronic slope	3.5	−0.2	7.1
eGFR-cystatin C at baseline[a]	< 60 ml/min/1.73m^2 (n=20)	Acute slope for Canagliflozin	−84.1	−137.9	−30.3
	(Canagliflozin: n=10, Control: n=10)	Acute slope for Control	40.3	−11.0	91.6
		Chronic slope for Canagliflozin	4.7	−0.1	9.6
		Chronic slope for Control	−2.6	−7.3	2.1
		Difference of acute slope	−124.4	−198.7	−50.1
		Difference of chronic slope	7.3	0.6	14.0
	≥ 60 ml/min/1.73m^2 (n=75)	Acute slope for Canagliflozin	−57.0	−82.5	−31.5
	(Canagliflozin: n=39, Control: n=36)	Acute slope for Control	−16.8	−45.0	11.4
		Chronic slope for Canagliflozin	−1.7	−4.2	0.7
		Chronic slope for Control	−5.5	−8.0	−3.0
		Difference of acute slope	−40.2	−78.2	−2.1
		Difference of chronic slope	3.8	0.3	7.3
Change in eGFR-creatinine from baseline to week 4[a]	< Median of the canagliflozin group (n=31)	Acute slope for Canagliflozin	−102.5	−137.1	−67.8
	(Canagliflozin: n=24, Control: n=7)	Acute slope for Control	−33.1	−97.7	31.6

(Median= −6.50%)		Chronic slope for Canagliflozin	1.5	−1.9	4.9
		Chronic slope for Control	0.4	−5.9	6.8
		Difference of acute slope	−69.4	−142.7	4.0
		Difference of chronic slope	1.1	−6.1	8.3
	≥ Median of the canagliflozin group (n=64)	Acute slope for Canagliflozin	−28.1	−58.5	2.4
	(Canagliflozin: n=25, Control: n=39)	Acute slope for Control	2.0	−23.7	27.6
		Chronic slope for Canagliflozin	−2.1	−5.1	0.9
		Chronic slope for Control	−5.8	−8.1	−3.5
		Difference of acute slope	−30.0	−69.9	9.8
		Difference of chronic slope	3.7	−0.1	7.5
Change in eGFR-cystatin C from	< Median of the canagliflozin group (n=34)	Acute slope for Canagliflozin	−129.9	−159.2	−100.5
baseline to week 4[a]	(Canagliflozin: n=24, Control: n=10)	Acute slope for Control	−75.3	−120.3	−30.2
(Median= −6.95%)		Chronic slope for Canagliflozin	1.6	−1.3	4.6
		Chronic slope for Control	−1.4	−6.0	3.2
		Difference of acute slope	−54.6	−108.4	−0.8
		Difference of chronic slope	3.0	−2.4	8.5
	≥ Median of the canagliflozin group (n=61)	Acute slope for Canagliflozin	−7.5	−35.4	20.4
	(Canagliflozin: n=25, Control: n=36)	Acute slope for Control	15.0	−10.6	40.6
		Chronic slope for Canagliflozin	−2.1	−5.3	1.2
		Chronic slope for Control	−5.8	−8.4	−3.1
		Difference of acute slope	−22.5	−60.4	15.3
		Difference of chronic slope	3.7	−0.5	7.9

Change in eGFR-creatinine from week 52 to 56[a,b] (Median= 4.57%)	< Median of the canagliflozin group (n=59) (Canagliflozin: n=24, Control: n=35)	Acute slope for Canagliflozin	−54.9	−90.2	−19.7
		Acute slope for Control	11.4	−18.0	40.8
		Chronic slope for Canagliflozin	−0.7	−3.8	2.4
		Chronic slope for Control	−4.9	−7.4	−2.3
		Difference of acute slope	−66.4	−112.3	−20.4
		Difference of chronic slope	4.2	0.2	8.2
	≥ Median of the canagliflozin group (n=33) (Canagliflozin: n=23, Control: n=10)	Acute slope for Canagliflozin	−62.6	−91.9	−33.3
		Acute slope for Control	−55.1	−102.5	−7.6
		Chronic slope for Canagliflozin	−0.6	−4.1	2.8
		Chronic slope for Control	−4.4	−9.7	0.8
		Difference of acute slope	−7.5	−63.3	48.3
		Difference of chronic slope	3.8	−2.5	10.1
Change in eGFR-cystatin C from week 52 to 56[a, b] (Median= 5.67%)	< Median of the canagliflozin group (n=53) (Canagliflozin: n=23, Control: n=30)	Acute slope for Canagliflozin	−33.2	−65.1	−1.3
		Acute slope for Control	7.7	−21.0	36.4
		Chronic slope for Canagliflozin	−0.6	−3.8	2.5
		Chronic slope for Control	−4.1	−6.9	−1.4
		Difference of acute slope	−40.9	−83.8	2.0
		Difference of chronic slope	3.5	−0.6	7.7
	≥ Median of the canagliflozin group (n=39) (Canagliflozin: n=24, Control: n=15)	Acute slope for Canagliflozin	−83.3	−116.7	−49.9
		Acute slope for Control	−20.0	−63.7	23.7
		Chronic slope for Canagliflozin	−0.7	−4.0	2.6
		Chronic slope for Control	−6.1	−10.3	−1.9
		Difference of acute slope	−63.3	−118.3	−8.4

		Difference of chronic slope	5.4	0.1	10.8
Use of ACE inhibitor or ARB at baseline	Yes (n=61) (Canagliflozin: n=28, Control: n=33)	Acute slope for Canagliflozin	−82.2	−113.4	−51.0
		Acute slope for Control	1.9	−27.5	31.2
		Chronic slope for Canagliflozin	0.4	−2.9	3.7
		Chronic slope for Control	−4.9	−8.0	−1.9
		Difference of acute slope	−84.1	−126.9	−41.2
		Difference of chronic slope	5.3	0.8	9.8
	No (n=34) (Canagliflozin: n=21, Control: n=13)	Acute slope for Canagliflozin	−34.3	−68.0	−0.6
		Acute slope for Control	−19.1	−63.6	25.3
		Chronic slope for Canagliflozin	−1.6	−4.4	1.3
		Chronic slope for Control	−4.6	−8.1	−1.1
		Difference of acute slope	−15.2	−71.0	40.6
		Difference of chronic slope	3.1	−1.5	7.6

The estimation of eGFR-cystatin C slopes was performed using a two-slope linear spline mixed effects model (with a knot at week 4) with unstructured covariance for random intercepts and slopes per participant. ACE, angiotensin-converting enzyme; ARB, angiotensin-receptor blocker; BMI, body mass index; eGFR, estimated glomerular filtration rate; HbA1c, glycated hemoglobin; UACR, urinary albumin-to-creatinine ratio.

[a]One participant, assigned to the control group, whose eGFR-creatinine and eGFR-cystatin C values were over 140 ml/min/1.73m^2 at baseline, weeks 4, 52, and 56 were excluded based on the predetermined Data Handling Rules for Statistical Analysis.

[b]Two and one participants assigned to the canagliflozin and control group, respectively, were excluded because both eGFR-creatinine and eGFR-cystatin C values were missing at either week 52 or 56, or both.

Supplementary Table S3: Least squares mean eGFR-creatinine slopes based on a two-slope linear spline mixed effects model using data from the same time points (weeks 0, 4, 12, 28, 44, and 52) as eGFR-cystatin C measurement.

eGFR-creatinine slopes	Estimate	95% CI	
	(ml/min/1.73m^2 per year)	Lower	Upper
Acute slope for Canagliflozin	−56.8	−76.3	−37.2
Acute slope for Control	−1.2	−21.8	19.4
Chronic slope for Canagliflozin	−0.3	−2.6	2.0
Chronic slope for Control	−3.8	−6.2	−1.5
Difference of acute slope (Canagliflozin − Control)	−55.6	−84.0	−27.2
Difference of chronic slope (Canagliflozin − Control)	3.5	0.2	6.8

eGFR, estimated glomerular filtration rate.

Supplementary Figure S1

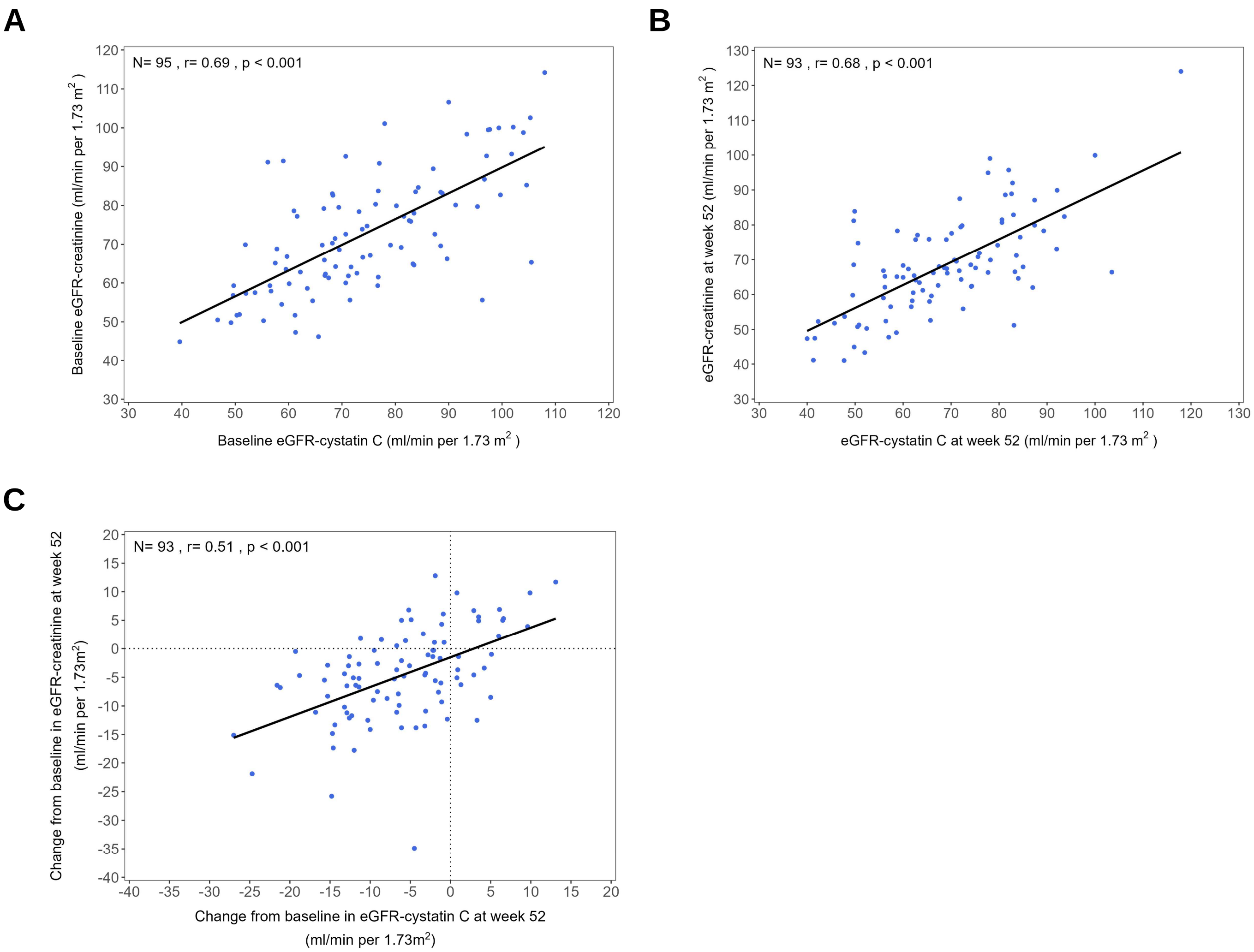

Supplementary Figure S1 | Correlations (Pearson's r) between eGFR-cystatin C and eGFR-creatinine.

Correlations between baseline eGFR-cystatin C and baseline eGFR-creatinine (*A*), eGFR-cystatin C and eGFR-creatinine at week 52 (*B*), and changes from baseline in eGFR-cystatin C and eGFR-creatinine at week 52 (*C*).

eGFR, estimated glomerular filtration rate.

Supplementary Figure S2

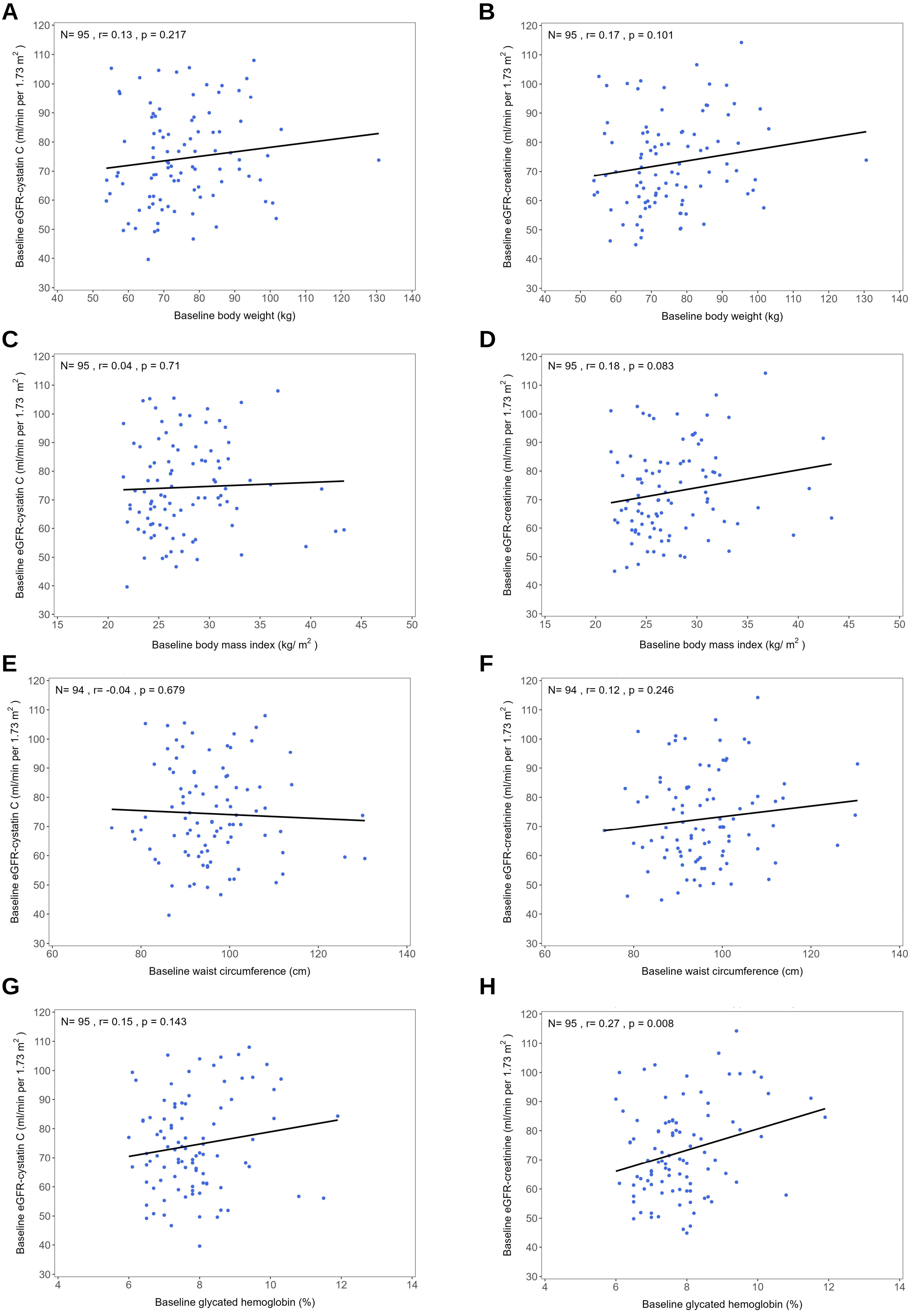

Supplementary Figure S2 | Correlations (Pearson's r) between eGFR (cystatin C and creatinine) and body weight, BMI, waist circumference, and HbA1c.
Correlations between baseline eGFR-cystatin C and body weight (*A*), baseline eGFR-creatinine and body weight (*B*), baseline eGFR-cystatin C and BMI (*C*), baseline GFR-creatinine and BMI (*D*), baseline eGFR-cystatin C and waist circumference (*E*), baseline eGFR-creatinine and waist circumference (*F*), baseline eGFR-cystatin C and HbA1c (*G*), and baseline eGFR-creatinine and HbA1c (*H*).
BMI, body mass index; eGFR, estimated glomerular filtration rate; HbA1c, glycated hemoglobin.

Supplementary Figure S3

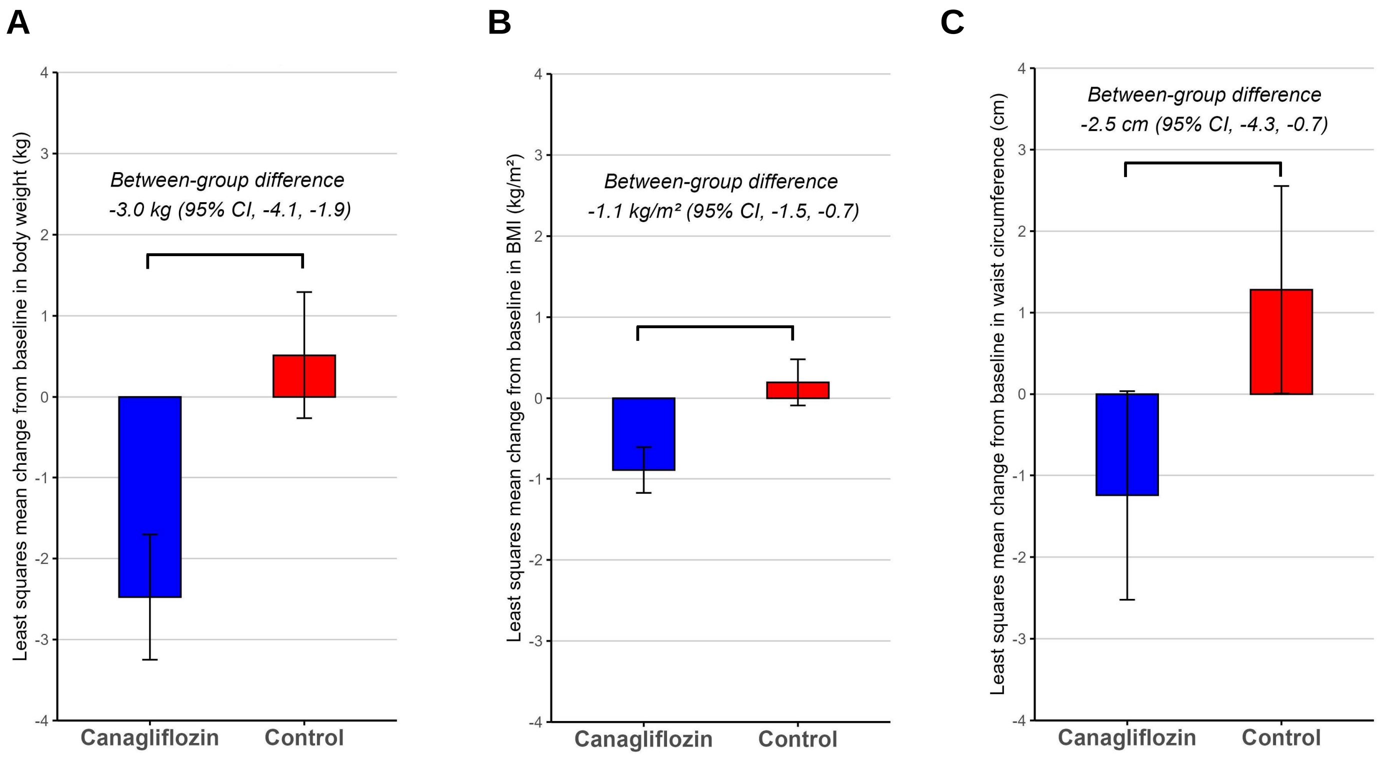

Supplementary Figure S3 | Least squares mean changes from baseline in parameters related to body composition at week 52.

Least squares mean changes from baseline in body weight (A), BMI (B), and waist circumference (C) at week 52. Error bars show 95% CIs.

BMI, body mass index.

Supplementary Figure S4

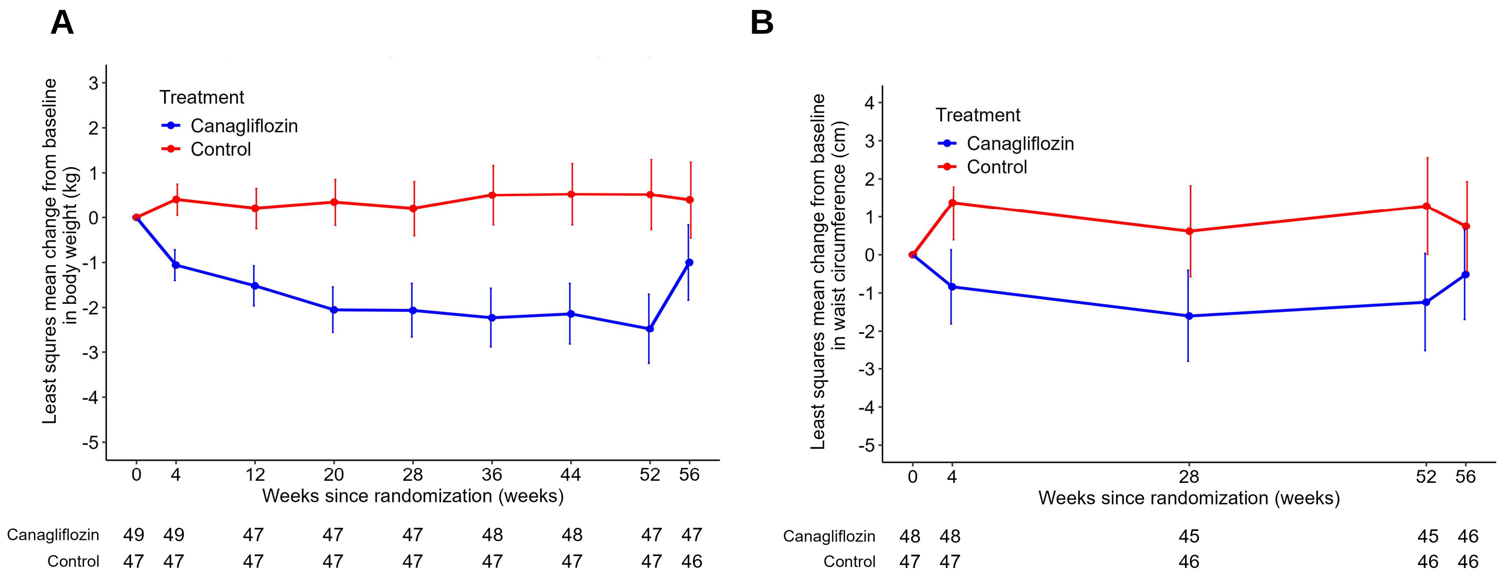

Supplementary Figure S4 | Least squares mean changes from baseline in parameters related to body composition over time.
Least squares mean changes from baseline in body weight (*A*) and waist circumference (*B*) over time. Error bars show 95% CIs.

Supplementary Figure S5

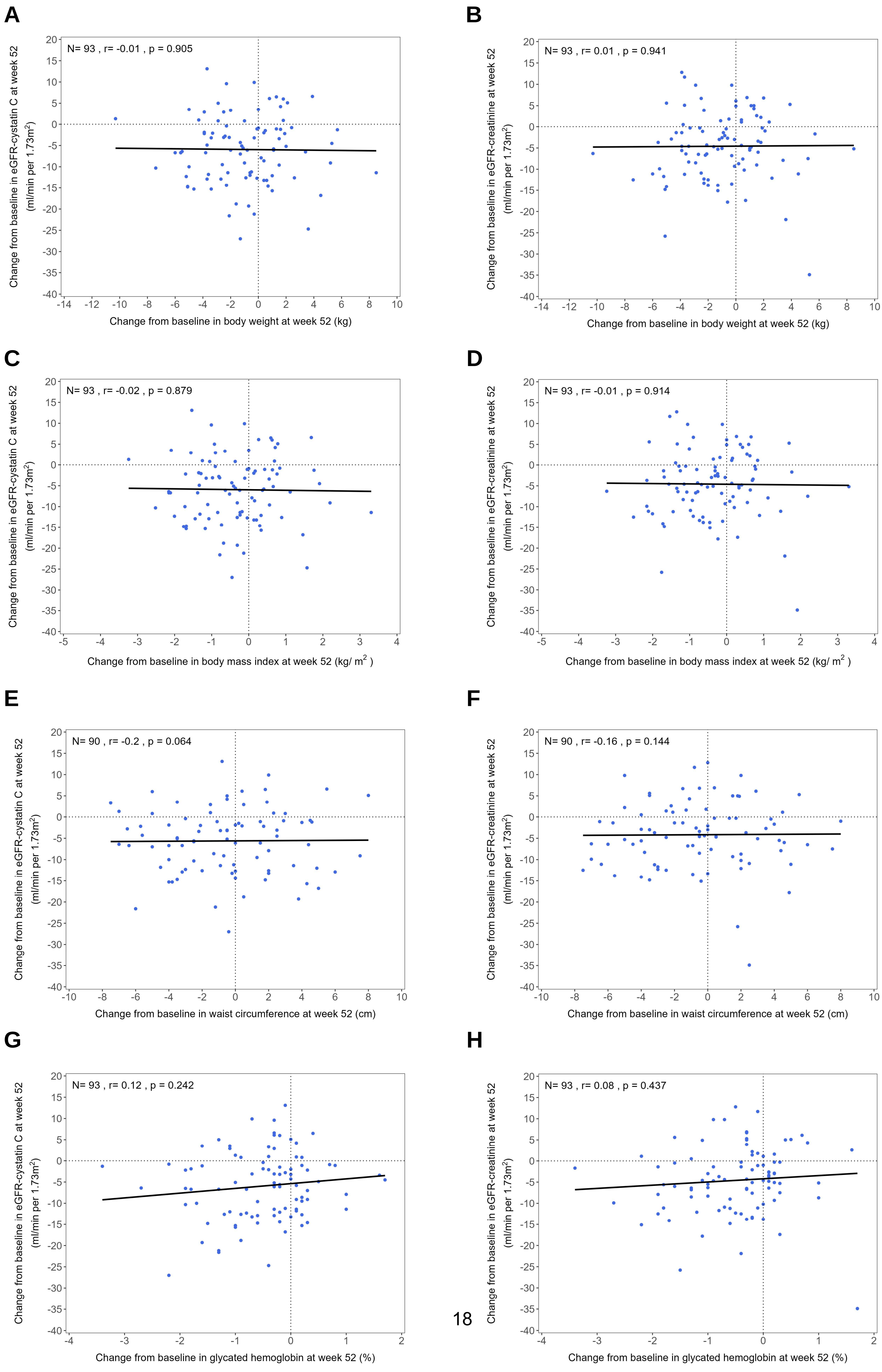

Supplementary Figure S5 | Correlations (Pearson's r) between changes from baseline in eGFR (cystatin C and creatinine) and in body weight, BMI, waist circumference, and HbA1c at week 52.

Correlations between changes from baseline in eGFR-cystatin C and body weight (A), in eGFR-creatinine and body weight (B), in eGFR-cystatin C and BMI (C), in eGFR-creatinine and BMI (D), in eGFR-cystatin C and waist circumference (E), in eGFR-creatinine and waist circumference (F), in eGFR-cystatin C and HbA1c (G), and in eGFR-creatinine and HbA1c (H), at week 52.

BMI, body mass index; eGFR, estimated glomerular filtration rate; HbA1c, glycated hemoglobin.

Supplementary Figure S6

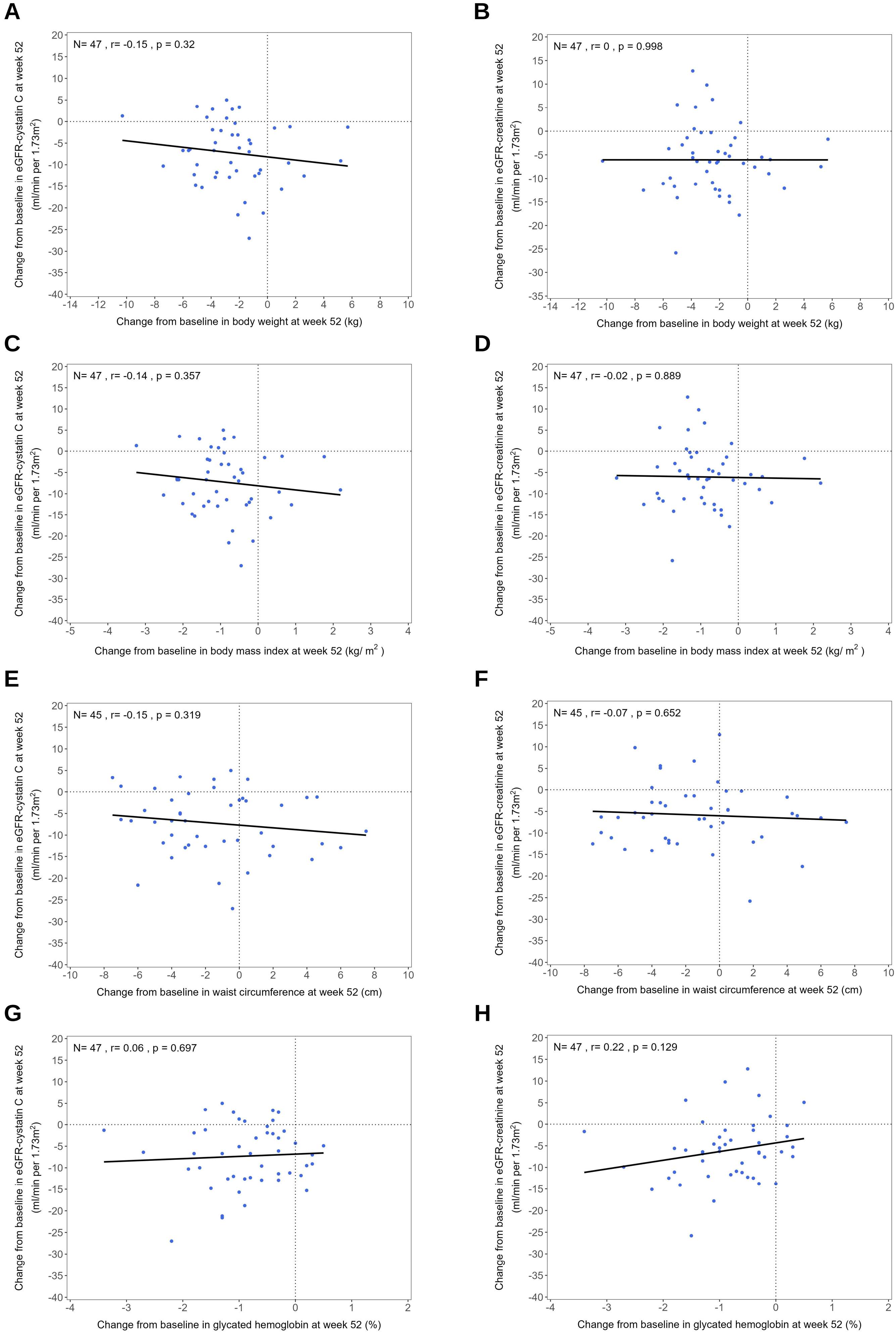

Supplementary Figure S6 | Correlations (Pearson's r) between changes from baseline in eGFR (cystatin C and creatinine) and body weight, BMI, waist circumference, and HbA1c at week 52 in the canagliflozin group.

Correlations between changes from baseline in eGFR-cystatin C and body weight (*A*), in eGFR-creatinine and body weight (*B*), in eGFR-cystatin C and BMI (*C*), in eGFR-creatinine and BMI (*D*), in eGFR-cystatin C and waist circumference (*E*), in eGFR-creatinine and waist circumference (*F*), in eGFR-cystatin C and HbA1c (*G*), and in eGFR-creatinine and HbA1c (*H*), at week 52 in the canagliflozin group.

BMI, body mass index; eGFR, estimated glomerular filtration rate; HbA1c, glycated hemoglobin.

Supplementary Figure S7

A

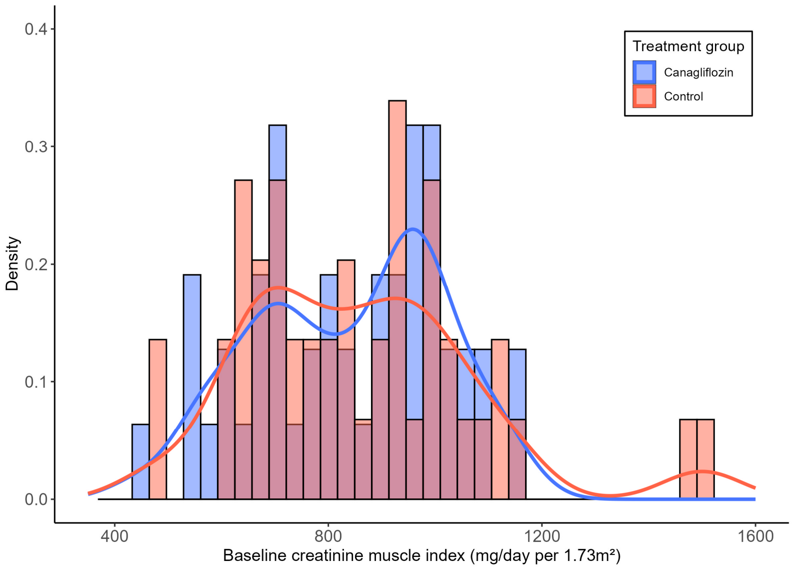

B

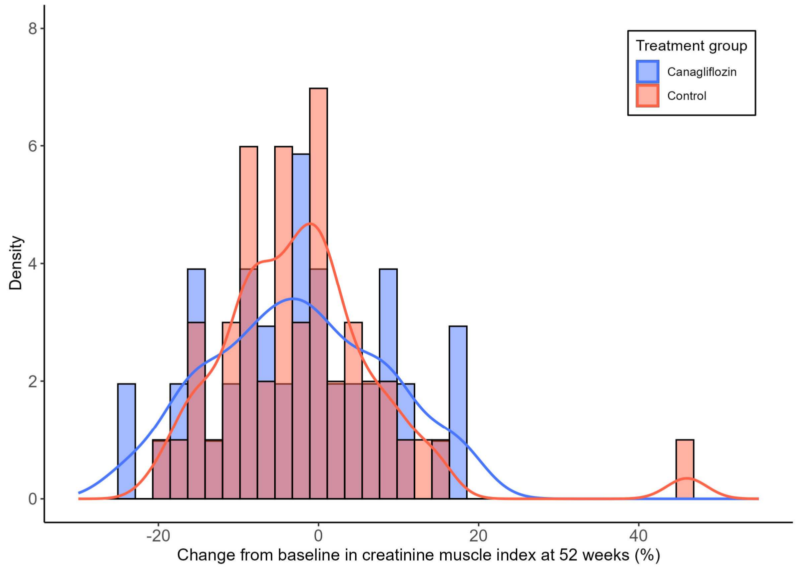

Supplementary Figure S7 | Comparison of the distribution of creatinine muscle index (CMI) in the canagliflozin and control groups.

The histograms for distribution of baseline CMI (*A*) and change from baseline in CMI at week 52 (*B*) in the canagliflozin and control groups. The solid lines show the kernel density estimate of the distributions.

CMI, creatinine muscle index.

Supplementary Figure S8

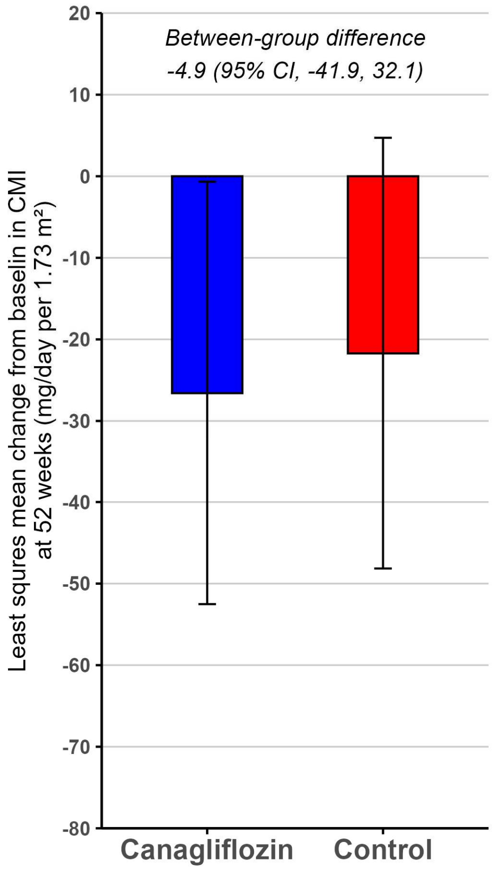

Supplementary Figure S8 | Least squares mean change from baseline in CMI at week 52.
Error bars show 95% CIs.
CMI, creatinine muscle index.

The CONSORT reporting checklist

For checking that reports of randomised trials can be understood and used by everyone

	Item Description	Location (or reason for not reporting)
**Title and Abstract**		
1a. Title	Identification as a randomised trial.	Previous paper (ref 2)
1b. Structured Abstract	Structured summary of the trial design, methods, results, and conclusions.	Page 4-5
**Open Science**		
2. Trial Registration	Name of trial registry, identifying number (with URL) and date of registration.	Page 8 and previous papers (refs 1 and 2)
Protocol and statistical analysis plan	Where the trial protocol and statistical analysis plan can be accessed.	https://jrct.mhlw.go.jp/latest-detail/jRCTs061180047
4. Data sharing	Where and how the individual de-identified participant data (including data dictionary), statistical code and any other materials can be accessed.	Page 19
5. Funding and Conflicts of Interest		
5a. Funding	Sources of funding and other support (eg, supply of drugs), and role of funders in the design, conduct, analysis, and reporting of the trial.	Page 19
5b. Conflicts of interest	Financial and other conflicts of interest of the manuscript authors.	Page 17-18
**Introduction**		
6. Background and rationale	Scientific background and rationale.	Page 6-7
7. Objectives	Specific objectives related to benefits and harms.	Page 7
**Methods**		
8. Patient and public involvement	Details of patient or public involvement in the design, conduct and reporting of the trial.	N/A
9. Trial Design	Description of trial design including type of trial (eg, parallel group, crossover), allocation ratio, and framework (eg, superiority, equivalence, non-inferiority, exploratory).	Page 7-8 and previous papers (refs 1 and 2)

10. Changes to trial protocol	Important changes to the trial after it commenced including any outcomes or analyses that were not pre-specified, with reason.	Page 16 and previous papers (refs 1 and 2)
11. Trial Setting	Settings (eg, community, hospital) and locations (eg, countries, sites) where the trial was conducted.	Page 7 and previous papers (refs 1 and 2)
12. Eligibility Criteria		
12a. Participants	Eligibility criteria for participants.	Page 7-8 and previous papers (refs 1 and 2)
12b. Other	If applicable, eligibility criteria for sites and for individuals delivering the interventions (eg, surgeons, physiotherapists).	N/A
13. Intervention and comparator	Intervention and comparator with sufficient details to allow replication. If relevant, where additional materials describing the intervention and comparator (eg, intervention manual) can be accessed.	Page 8 and previous papers (refs 1 and 2)
14. Outcomes	Prespecified primary and secondary outcomes, including the specific measurement variable (eg, systolic blood pressure), analysis metric (eg, change from baseline, final value, time to event), method of aggregation (eg, median, proportion), and time point for each outcome.	Previous papers (refs 1 and 2)
15. Harms	How harms were defined and assessed (eg, systematically, non-systematically).	Previous paper (ref 2)
16. Sample Size		
16a. How sample size was determined	How sample size was determined, including all assumptions supporting the sample size calculation.	Previous papers (refs 1 and 2)
16b. Interim analyses and stopping criteria	Explanation of any interim analyses and stopping guidelines.	Previous papers (refs 1 and 2)
17. Randomisation		
17a. Sequence Generation	Who generated the random allocation sequence and the method used.	Previous papers (refs 1 and 2)
17b. Type of Randomisation	Type of randomisation and details of any restriction (eg, stratification, blocking, and block size).	Previous papers (refs 1 and 2)
18. Allocation concealment mechanism	Mechanism used to implement the random allocation sequence (eg, central computer/telephone; sequentially numbered, opaque, sealed containers), describing any steps to conceal the sequence until interventions were assigned.	Previous papers (refs 1 and 2)

19. Implementation	Whether the personnel who enrolled and those who assigned participants to the interventions had access to the random allocation sequence.	Previous papers (refs 1 and 2)
20. Blinding		
20a. Who was blinded	Who was blinded after assignment to interventions (eg, participants, care providers, outcome assessors, data analysts).	N/A
20b. How blinding was achieved	If blinded, how blinding was achieved and description of the similarity of interventions.	N/A
21. Statistical methods		
21a. Comparing groups	Statistical methods used to compare groups for primary and secondary outcomes, including harms.	Previous paper (refs 1 and 2)
21b. Definition of who is included in each analysis	Definition of who is included in each analysis (e.g., all randomised participants), and in which group.	Page 9 and previous papers (refs 1 and 2)
21c. Missing Data	How missing data were handled in the analysis.	Previous paper (ref 2)
21d. Additional Analyses	Methods for any additional analyses (eg, subgroup and sensitivity analyses), distinguishing pre-specified from post hoc.	Page 9-10 and previous papers (refs 1 and 2)
22. Participant flow, including flow diagram		
22a. Participant Numbers	For each group, the numbers of participants who were randomly assigned, received intended intervention, and were analysed for the primary outcome.	Previous paper (ref 2)
22b. Losses and exclusions	For each group, losses and exclusions after randomisation, together with reasons.	Previous paper (ref 2)
23. Recruitment		
23a. Dates	Dates defining the periods of recruitment and follow-up for outcomes of benefits and harms.	Previous paper (ref 2)
23b. Reasons for stopping recruitment	If relevant, why the trial ended or was stopped.	N/A
24. Intervention and comparator delivery		
24a. As Administered	Intervention and comparator as they were actually administered (eg, where appropriate, who delivered the intervention/comparator, whether participants adhered, whether they were delivered as intended (fidelity)).	Previous paper (ref 2)

24b. Concomitant Care	Concomitant care received during the trial for each group.	Previous papers (refs 1 and 2)
25. Baseline Data	A table showing baseline demographic and clinical characteristics for each group.	Table 1 and previous papers (refs 1 and 2)
26. Numbers analysed, outcomes, and estimation	For each primary and secondary outcome, by group:<br>• the number of participants included in the analysis.<br>• the number of participants with available data at the outcome time point.<br>• result for each group, and the estimated effect size and its precision (such as 95% confidence interval).<br>• for binary outcomes, presentation of both absolute and relative effect size.	Previous paper (ref 2)
27. Harms	All harms or unintended events in each group.	Previous paper (ref 2)
28. Ancillary Analyses	Any other analyses performed, including subgroup and sensitivity analyses, distinguishing pre-specified from post hoc.	Page 9-10 and previous paper (ref 2)
**Discussion**		
29. Interpretation	Interpretation consistent with results, balancing benefits and harms, and considering other relevant evidence.	Page 14-17
30. Limitations	Trial limitations, addressing sources of potential bias, imprecision, generalisability, and, if relevant, multiplicity of analyses.	Page 16
